# Sweat, Skepticism, and Uncharted Territory: A Qualitative Study of Opinions on Data Sharing Among Public Health Researchers and Research Participants in Mumbai, India

**DOI:** 10.1177/1556264615592383

**Published:** 2015-07

**Authors:** Ketaki Hate, Sanna Meherally, Neena Shah More, Anuja Jayaraman, Susan Bull, Michael Parker, David Osrin

**Affiliations:** 1 Society for Nutrition, Education and Health Action, Mumbai, India; 2 University of Oxford, UK; 3 UCL Institute for Global Health, London, UK

**Keywords:** data sharing, poverty areas, Mumbai, India, ethics

## Abstract

Efforts to internalize data sharing in research practice have been driven largely by developing international norms that have not incorporated opinions from researchers in low- and middle-income countries. We sought to identify the issues around ethical data sharing in the context of research involving women and children in urban India. We interviewed researchers, managers, and research participants associated with a Mumbai non-governmental organization, as well as researchers from other organizations and members of ethics committees. We conducted 22 individual semi-structured interviews and involved 44 research participants in focus group discussions. We used framework analysis to examine ideas about data and data sharing in general; its potential benefits or harms, barriers, obligations, and governance; and the requirements for consent. Both researchers and participants were generally in favor of data sharing, although limited experience amplified their reservations. We identified three themes: concerns that the work of data producers may not receive appropriate acknowledgment, skepticism about the process of sharing, and the fact that the terrain of data sharing was essentially uncharted and confusing. To increase data sharing in India, we need to provide guidelines, protocols, and examples of good practice in terms of consent, data preparation, screening of applications, and what individuals and organizations can expect in terms of validation, acknowledgment, and authorship.

With an estimated population of 1.2 billion, a history of science going back at least three millennia, a patchwork of groups at different stages of the demographic, epidemiologic, and nutrition transitions, and highly developed educational and health sectors, India is a major producer of biomedical research data. The potential uses of these data are, however, often untapped as they reside in diverse, largely unconnected, caches. The practice of data sharing for biomedical research has been uncommon in India.

There is debate about whether data sharing should be an ethical imperative. One position is that it is a public good. It allows scrutiny and alternative analyses, makes researchers more accountable, contributes to public faith in the pursuit of science, raises the possibility of answering new questions, and reduces duplication and the burden on participants (Bersoff & Dawes, 1992; Bishop, 2009; Budin-Ljøsne et al., 2014; Chandramohan et al., 2008; Larson, 2013). Another position is that data sharing cannot be mandatory because of concerns about confidentiality and the relationship between researchers and participants, consent, transferability, logistic issues, and potential misuse (Bersoff & Dawes, 1992; Cooper, 2007; Greenhalgh, 2009).

The arguments are being rehearsed amid an international shift toward inclusion of data sharing in routine research governance. This shift has been driven by funding bodies (Bishop, 2009; Pearce & Smith, 2011; Pisani & AbouZahr, 2010; Rani, Bekedam, & Buckley, 2011; Rani & Buckley, 2012), and, although largely consensual, its articulation of data sharing as “ethical, efficient and equitable” (Wellcome Trust, 2011) has not gone without criticism (Dawson & Verweij, 2011; Greenhalgh, 2009). Few policies have so far addressed data sharing in India. At macro level, the Government of India Department of Science and Technology has developed a National Data Sharing and Accessibility Policy (Version 2.1, 2013: http://www.dst.gov.in/nsdi.html) under which publicly funded data generated by government agencies are to be made available to intra- and inter-governmental agencies. An obvious example is the Census of India (http://censusindia.gov.in). A draft bill on the right to privacy is also under consideration by the Ministry of Home Affairs. At meso level, the Indian Council of Medical Research (2006) ethical guidelines for biomedical research on human participants point to researchers’ responsibility to envision any future use of data for secondary purposes. Paragraphs on data sharing also appear in the ethical guidelines produced by the National Committee for Ethics in Social Science Research in Health (2000). At micro level, a number of organizations have developed data sharing policies. Examples include the Centre for Chronic Conditions and Injuries, affiliated to the Public Health Foundation of India, and Sangath, a non-profit organization. Funder mandates for data sharing have also gained momentum. For example, guidelines exist for accessing data from work by the National AIDS Research Institute that has been funded by the Bill & Melinda Gates Foundation (see, for instance, http://www.nari-icmr.res.in/pdf/IBBA/Data-access-Information.pdf).

Against this backdrop, the Society for Nutrition, Education and Health Action (SNEHA), a non-governmental organization, works to improve health in urban informal settlements (slums) in Mumbai, India. The context in which data might be shared is unusual—but representative of changes across the non-government sector—in that the organization combines research with action. Primarily used for program evaluation, data are collected from women and children in informal settlements. They describe assets, education, family planning, maternity experience, use of health care providers, agency, mortality and morbidity, nutrition, and violence against women and children. As such, they are of interest to other non-governmental organizations, government agencies, activists, and social and biomedical scientists.

As a member of the collaboration on developing ethical data sharing processes for public health data in research, we sought to identify features of ethical data sharing practice in the context of research involving women and children in informal settlements (Cheah et al., 2015; Denny, Silaigwana, Wassenaar, Bull, & Parker, 2015; Jao et al., 2015; Merson et al., 2015; Parker & Bull, 2015). Our specific objectives were to examine stakeholders’ understandings, concerns, and hopes about what would happen to data and their views on what might constitute good data sharing practice; to identify models of data sharing and governance currently in use; to examine contextual considerations affecting data sharing processes; to identify perceived principles of good practice in data sharing; and to consider suitable methods of developing appropriate data sharing processes (Bull, Cheah, et al., 2015; Bull, Roberts, & Parker, 2015).

## Method

### Setting

Within the remit of the collaborative study, we aimed to interview people drawn from two pools: employees or participants in research conducted by SNEHA, augmented by researchers from other organizations with experience of either data sharing or the ethical issues around it. We tried to cover a spectrum, from community members who had taken part in studies, through field data collectors, data entry officers, analysts, and research designers, to organizational executives. Many of the data collected by SNEHA relate to health issues for women and children in urban informal settlements. Around 40% of Mumbai’s residents live in informal settlements with attendant constraints on finances, space, water and sanitation, and access to adequate health care (Officer of the Registrar General & Census Commissioner, & Director of Census Operations Maharashtra, 2011). These constraints lead to aspirations for themselves and their communities that might inform interviewees’ responses to questions about data sharing. There is substantial diversity within informal settlements in terms of longevity, place of origin, religion, language, and cultural mores. A minority of women are involved in formal sector work, and many have limited control over resources and limited agency. Daily life involves a negotiation between the opportunities of urban modernity and the persistence of traditional norms.

### Interviewers

Two full-time female interviewers (K.H. and S.M.) collected the data through semi-structured interviews and focus group discussions. Both have postgraduate degrees and experience of collecting and analyzing qualitative data. All the participants were familiar with the organization and its work, if not with the interviewers themselves. More than half of them worked with the organization, and all but one of the others were recruited through the authors’ personal and professional contacts or those of community outreach workers.

### Participant Selection

A mix of purposive and convenience sampling was used. Participants for semi-structured interviews were selected for their level of familiarity with data sharing. Table 1 summarizes the sample. It was easy to find people within the organization who had no experience of data sharing, but interviews with experienced people tended to be outside the organization. We grouped interviewees by their level of involvement in the process and ethics of research, as senior, mid-level, and junior biomedical and social science researchers, program implementers, policymakers, and ethicists. The focus groups were conducted with field-workers involved in community mobilization and with women from the communities in which the organization worked. We contacted interviewees by email and telephone, field-workers by telephone or face-to-face, and community members through field-workers who already knew them. Two potential interviewees did not respond to email invitations.

**Table 1.** Participants.

Stakeholders	SNEHA	External	Data collection method
Managers or decision makers	2	0	Interview
Project implementers	2	0	Interview
Senior researchers	1	4	Interview
Mid-level researchers	3	0	Interview
Junior researchers or assistants	6	1	Interview
Ethics committee members	0	3	Interview
Field data collectors	24	0	Focus group discussion
Community members	0	20	Focus group discussion
Total	38	28	66

*Note.* SNEHA = Society for Nutrition, Education and Health Action.

When not responding to specific examples posed by interviewers and focus group facilitators, it is likely that participants associated with SNEHA (either as researchers or as community members) would have been thinking about research that involved collection of interview data about maternal and newborn health, nutrition, sexual and reproductive health, or gender-based violence. Ethicists were drawing on their experience of social science research, and external senior researchers were involved in descriptive studies, laboratory work, and clinical trials on communicable diseases and mental health.

### Interview Arrangements

We interviewed participants at a time and place convenient to them: one at home and the rest at their workplaces. Two focus group discussions with field-workers were conducted at program offices and two with community members at outreach centers. When interviewees were outside Mumbai, we communicated through Skype and used third-party software to record the interviews (www.ifree-recorder.com). Interviews were in English, Hindi, or Marathi, depending on interviewees’ proficiency and comfort. With help from a participant information sheet, the interviewers explained the reasons for the study and the aims of the interview. K.H. and S.M. led interviews alternately—one as primary interviewer and the other as an observer—apart from three solo interviews. They were also co-facilitators for the focus group discussions. We held two focus groups with field-workers and two with community members. Three field-workers attended the community discussions because we thought that their presence would reassure the participants. They had similar backgrounds and we did not think that their presence would hinder open discussion.

### Interview Content

Topic guides were based on a structure agreed across the collaboration, reordered on the basis of experience and natural flow. We began by asking interviewees about their research experience and areas of interest, organizational roles, and history of collaboration. We asked what they understood by data, sensitive data, and data sharing. We asked about their experiences of data sharing—access or provision—and agreements and obstacles to it, their ideas of the advantages and disadvantages, the kinds of data that should or should not be shared, and their views on current policies, barriers, and recommendations for good practice. We explored their views on researchers’ responsibilities and how participants’ interests could be protected during the process and on data sharing across cultures and research contexts, particularly on sharing data between higher- and lower-income countries. We asked about existing consent processes, their views on broad and explicit consent, and any constraints that participants might put on secondary uses of their data. We concluded by asking for their views on sharing data from the interview itself.

For focus groups, we developed a series of scenarios that drew on their previous contributions to research. We began by describing a scenario in which data on the prevalence of malnutrition and tuberculosis might be shared with other entities. These entities were presented in steps, beginning with local or foreign students and broadening organizationally and geographically to researchers and national and international organizations. We also presented scenarios with increasing sensitivity, for example, information about family planning or violence in the case of a woman who might not want her family to know about it.

Interviews and focus group discussions were audio-recorded and transcribed verbatim. Discussions in Hindi and Marathi were subsequently translated into English. Field notes described observations of participant behavior and responses. Transcripts were given to participants who were interested in reviewing them, and we are not aware of any concerns. Interviews lasted an average 51 min (range: 14-91). Focus group discussions lasted 42 min (range: 28-60).

Data were collected in two phases. In the first, 12 interviews and two focus group discussions were conducted and coded broadly. In the second, we tried to sample and probe on the basis of information gaps and emerging themes. We discussed data saturation continually from the latter part of the first phase and felt that we were accruing no new responses by the final interviews.

### Theoretical Framework

We used framework analysis because we already had an idea of the terrain of data sharing, a set of a priori questions, specific objectives for outputs (recommendations for ethical considerations in data sharing policy and practice), and an agreed sample size and timeline for deliverables (Ritchie & Spencer, 1994; Srivastava & Thomson, 2009). Because the interviews followed a structured sequence and addressed issues that we already thought might be important, we agreed across the collaborating groups to begin with a list of general coding categories. In assigning information to these categories, we developed a series of subheadings (nodes), including new subheadings that reflected interviewees’ views and allowed us to think about themes that emerged from the data (Lacey & Luff, 2007; Pope, Ziebland, & Mays, 2000; Smith & Firth, 2011).

### Analysis

Transcripts were imported into NVivo 10 (www.qsrinternational.com), in which coding and analysis were done. The coding tree was developed collectively in sessions during which pseudonymized transcripts were projected. Beginning with the consensual tree for the multisite study, we added sub-nodes without disrupting the general framework of parent nodes. From halfway through the first phase, initial coding was done by K.H. and S.M., after which it was reviewed by the other authors. Nodes were annotated with descriptions, and a second round of coding reviewed the exhaustiveness and utility of the coding tree. We mapped concepts and explanations through several iterations (Attride-Stirling, 2001) and discussed them with the wider collaborative group at an international meeting, through a shared electronic repository, and at fortnightly teleconferences.

### Ethics Review

The multisite study was approved by the Oxford Tropical Research Ethics Committee (OxTREC 1051-13). The India study was approved by the Multi-Institutional Ethics Committee, Mumbai.

## Results

We collected data from 66 people: 22 in interviews and 44 in four focus group discussions. Thirty-eight people worked with the organization, 8 worked with other biomedical or social science research organizations or ethics committees, and 20 were female community members. We expected 22 interviewees to have experience of data sharing, but only 8 were familiar with the practicalities. None had accessed other researchers’ datasets. In two cases, academics contacted because of their extensive research experience turned out not to have shared or accessed data.

### Ideas About Data in General, Sensitive Data, and Sharing

Most interviewees said that data were information that helped to understand the phenomenon being studied. They included—but were not limited to—demographic and household details, images, videos, and medical records, and they could be quantitative or qualitative. Almost all data could be sensitive, which made it important to understand the context in which they had been collected. Information that could harm an individual, community, or organization was considered particularly sensitive. Examples given included HIV status, history of abuse, sexual behavior, family planning, and medical and financial records. Although some interviewees were skeptical about sharing such data, most said that they could be shared if anonymity was guaranteed.

We asked about sharing, not just of data, but in a more general sense. Interviewees said that sharing varied culturally and with social position. Two said that Indians were more likely to share than people from the North who were more cautious for cultural reasons and were more aware of the idea of confidentiality. Members of less affluent groups were more likely to share because of a degree of tolerance of lack of privacy, and some said that small, close-knit communities tended to see sharing as a cultural good.

### Benefits of Data Sharing

Interviewees described the potential benefits of data sharing in four broad ways: it generated evidence, increased transparency, avoided duplication of effort, and encouraged learning. Knowledge built through data sharing allowed policymakers to make informed, occasionally groundbreaking, decisions and could contribute to changes in policy and enable rapid action on public health issues.

. . . It is by data sharing that you work out where the nexuses of resistance to artemisinins are . . . diseases like SARS and MERS . . . triggered a lot of data sharing initiatives . . . to track these emerging epidemics. (Senior Researcher, IN-SR-I-81, male)

Data sharing increased the transparency and validity of inferences and prevented misuse. It saved resources and respected participants’ privacy and dignity.

. . . You are in this . . . dilemma of saying, “how do I as a researcher go to that person and . . . ask the very same thing which a person just like me had just asked. . . . When I can easily talk to that person and get that data.” (Ethicist, IN-RC-I-34, male)

It also helped students and researchers learn, sometimes by connecting people internationally. New findings, research questions, and funding might emerge, and analyses might achieve more than researchers would otherwise have managed. “. . . Other researchers may have elevated ways of looking at that data that the original investigators may not have actually thought about, or don’t have the time to address” (Senior Researcher, IN-SR-I-73, male).

### Harms of Data Sharing

Interviewees saw potential disadvantages in two areas: misuse and harms to participants. They objected to the use of data for market research or commercial activity, and the interests of sharer and accessor had to be aligned because data could be manipulated to tell a certain story. The aims of the accessor might not be clear, and there might be no way of constraining interpretation in an environment of competing ideologies. “The requester has never ever been very transparent and upfront and never given us enough comfort for us to say, yes, fine, we will go and share the data” (Executive, IN-MP-I-93, female). Data use might also result in a pejorative presentation of a community, despite anonymization.

Interviewees were particularly concerned that participants would not benefit from data sharing. They also worried that participants might respond less freely if they knew that their data were going to be shared.

When data is collected from participants, especially sensitive data, and participants are informed that their data may be shared with another entity, the participant may worry about her information being shared and there’s a possibility that she provides inaccurate data, thus jeopardizing the process of data collection. (Field-Worker, IN-FS-G-76, female)

### Barriers

Perceived barriers to data sharing fell into three general groups: limited precedent, uncertainty about user agendas, and the work involved in enabling sharing. Most respondents had minimal experience of sharing data or accessing it from other sources. They were aware of few precedents and pointed to a deficiency of protocols and structures by which they might be guided, against a background of organizational and individual competition with little tradition of institutional collaboration. “I think in India the whole concept of ownership, authorship itself is very broad . . . I don’t think there is an environment where people would feel very comfortable to share data” (Senior Researcher, IN-SR-I-95, female).

Ethics committees were sensitive to the concerns and effects of data sharing, but an ethicist said that institutions conducting research tended to set up Institutional Ethics Committees at the behest of funders rather than in their own interests. Sharing might come with no personal or institutional benefit, and mistrust of other researchers was common.

There is a lot of fear that . . . they will be robbed of their data . . . In India, unfortunately, we don’t have a strong culture of integrity in our institutions. There is no good system to prevent misconduct in research, so people who are powerful in the institution, they exploit people who are weak. (Ethicist, IN-RC-I-51, male)

The effort invested in data collection, and a desire for work to be recognized and appreciated, featured in several interviews. “I have collected the data. I have taken the effort in creating this protocol and going through all the procedures and then interacting with the patient . . . why should I just give it to you free?” (Ethicist, IN-SR-I-58, male). Interviewees at all levels of experience, but particularly those closer to the challenges of data collection, were often resistant to making life easier for other researchers.

But it’s not right that I take everything from my home and give it to someone else, isn’t it? . . . I have handled all of this like my own home, I collected all this data and then someone just came in and asked for my data. They can collect their own data, right? (Data Operative, IN-YN-I-42, female)

This did not rule out sharing if the accessor put some work in.

I feel that if we give them ready-made data then they will not value it as much as they should. If we let them know about the efforts we take for them and how we work so hard . . . (Field-Worker, IN-FS-G-63, female)

Data would also be context-specific, and the accessor might not appreciate their nuances. “If we share with them and have a discussion with them then we will feel good, and it will be easier for them to take the data too because we can then tell them in a better way” (Field-Worker, IN-FS-G-63, female).

Substantial work was required to prepare data for sharing and to administer the process.

It requires expertise and resources. As of now it is a big disadvantage that if I start pushing it in the institution some researchers may have to spend a lot of time there when they may be writing new proposals and getting more money for the institution; and the market setting now . . . everything is determined by your capacity to generate resources. (Ethicist, IN-RC-I-51, male)

Qualitative data presented additional problems, including the concern that preparation would require an understanding of the presentational aspects of datasets, clean data, codebooks, and intelligible labeling.

. . . There are many, many reasons I might not want to embark on this ship of data sharing. One of them might be that there’s work involved, it’s going to take time, I’ve got to clean the datasets and I’ve got to respond to their applications for it, I’ve got to put in a data sharing policy, I’ve got to convene that little committee that I’m talking about . . . All of this is more work . . . If I don’t have to do this, why would I start? (Senior Researcher, IN-SR-I-81, male)

Beside the amount of work that would be necessary—in an underfunded, time-poor environment—interviewees’ concern about the quality of their own data was a potential barrier to sharing them. Scrutiny of one’s data by a third party might be beneficial in terms of outputs, but it would bring with it potentially debilitating concerns about exposure of one’s research quality. “The data is uncleaned, unorganized; hence it is difficult to understand by the accessor and the sharer may forget previously done analysis. All this may cause embarrassment to the original researcher and prevent data sharing” (Senior Researcher, IN-SR-I-81, male).

### Obligations and Responsibilities

Interviewees at all levels said that ensuring consent and confidentiality were researchers’ most important responsibilities. This was a particular concern of field-workers who collected data. Other concerns included the need to maintain trust and to give something back to participants. Maintenance of confidentiality revolved around anonymization of datasets, and interviewees often questioned the possibility of sharing qualitative data because they were more difficult to anonymize than quantitative datasets. Field-workers said that researchers were responsible for assuring participants that their trust in them and the organization was sustained.

I believe that trust shouldn’t be broken because we won’t give anyone any information if they come to our home, but we go to their homes and they give us their information. This means that they trust us, they think that we are like them and that we will keep their information to ourselves. (Field-Worker, IN-FS-G-76, female)

Although both data sharers and accessors were obliged to maintain confidentiality, interviewees said that the onus of doing so rested largely with sharers because of their relationship with participants. Because of the trust relationship, researchers were indebted to participants. Field-workers suggested that access to data might be conditional on subsidized services, health care interventions, or provision of medicines: “If they are going to come up with some solution because of data sharing, then great, but if they are just going to write about it . . . then what’s the use?” (Field-Worker, IN-PC-G-84, female). Researchers also talked about giving something back to participants, but their suggestions were less specific. Research participants themselves were keen for their communities to benefit—“Data sharing is acceptable if the community benefits from it; there is no point in merely writing about issues” (Community Member, IN-PC-G-12, female)—but some participants were not set on this quid pro quo and said that data could be shared without direct community benefit: “Everything is not done to gain or benefit from it; it is sometimes [just] information . . . if they are writing about it, others will come to know . . .” (Community Member, IN-PC-G-12, female). Nevertheless, when community members were discussing data about burdens such as tuberculosis and cancer, they tended to say that data should be channeled toward direct intervention.

### Prerequisites for Data Sharing

A few interviewees took matter-of-fact positions, saying that once primary results were published, the data were in the public domain. Most, however, were more comfortable if the accessor represented an organization. “It is acceptable to let reputed educational institutes and international NGOs access data, but not any lay person” (Junior Researcher, IN-JR-I-83, female). Alignment of interests was again an issue: “. . . Only those who work on health . . . and not just any organization, but the one that is actually doing good work in health” (Field-Worker, IN-FS-G-63, female). Participants were wary of sharing data with commercial entities and favored “world-class researchers” or institutions such as the World Health Organization (WHO) and “eminent scholars.” The justification was that this would lead to “the betterment of the participants from the community” (Field-Worker, IN-FS-G-63, female), but it also influenced credibility and presumed competence: “Reputable organizations tend to have data sharing policies in place along with the necessary experience in conducting research” (Junior Researcher, IN-JR-I-28, female). Familiarity with organizations increased their credibility:

I know the people . . . and they are the sort of people that in the past have asked us to share data with them, and I know what the endeavor that they are engaged in is . . . and I know what the outputs will be: they will be high. There will be either a report or multiple papers in health journals that are peer reviewed . . . . (Senior Researcher, IN-SR-I-81, male)

Some participants said that data could be shared with students, while others said that students should make the effort to collect primary data for their own education.

Interviewees wanted to retain a stake in the data, but their uncertainty about how this might be done was a substantial disincentive to sharing. Most said that the sharer had to know the accessors’ intentions, check the relevance of data, and seek clarification. Transparency of objectives and use were mentioned repeatedly.

Another thing would be that people who live in an informal settlement in Mumbai are having a very tough life and they have invested time and given you information and nothing happens for them, but other people . . . get promoted and an industry swarms around, and this person is . . . providing the fuel for the industry without getting any of the product . . . and I think that would be . . . inequitable. (Senior Researcher, IN-SR-I-81, male)

Most participants expressed a preference for managed rather than open access to data.

. . . In practice I think you would definitely put procedures in place to make sure that the sharing was managed rather than open . . . and I think that’s what we’ve seen they do . . . at the major sharing portals.” (Senior Researcher, IN-SR-I-81, male)

Transparency might be assured by imposing conditions. “. . . And then you get adequate credit, your feedback is taken, your input is taken because at the end of the day you are generating the data, you also have a view” (Executive, IN-MP-I-93, female).

### Governance and Policy

Establishing governance and policy structures within an organization was an important step for most participants. Sharing should be contingent on permission from organizational managers and funders, memoranda of understanding, consent from participants, sharing output prior to publication, acknowledgment of field investigators and sharers, anonymization, and removal of sensitive data. Data sharing could be overseen by a committee whose members might include a senior executive who could weigh harms and benefits from the organizational perspective, an internal researcher experienced in publication and data sharing, stakeholders involved in the research process, a representative of the community from whom data were collected, and an external researcher or ethicist.

The committee would be the gatekeeper for managed access to data. It would “. . . enter into dialogue . . . essentially, the organization would go proxy for the participant who’s provided the data . . .” (Senior Researcher, IN-SR-I-81, male). The development of a memorandum of understanding would be an opportunity for dialogue:

. . . which data is being shared, how is it being shared, what variables are being shared, how the data’s going to be presented, who they can share that data with . . . clearly stipulate what they can do with that data . . . how are they going to store it or return it . . . destroy it . . . (Mid-Level Researcher, IN-KR-I-23, female)

As well as stipulating any monetary benefit for sharing data, the agreement would clarify ownership and authorship rights. Most interviewees said that ownership and recognition rested with the organization that had collected the data, rather than with the participants themselves. This included a range of individuals involved in the study, and they and the organization should be acknowledged in accordance with the use of the data. Some field-workers thought that research funders might also claim ownership. If a large quantum of data was used, if the accessor based a publication solely on the sharers’ data, or if the sharer had made a substantial contribution to the analysis or draft, authorship could be claimed. Some suggestions were more hopeful. For example, an executive said that blame for negative repercussions should be shared, and several interviewees suggested some form of monitoring to protect participants, researchers, and organizations from misinterpretation or misuse of data. Options included involving a team member in the publication process and reviewing drafts before publication.

### Broad, Middle, and Explicit Consent

We discussed three types of consent. Broad consent implied that participants were told that their data might be shared after use in the index study and that they would not be contacted for an opinion on sharing. The research organization would generally stand proxy for the participant in deciding whether sharing was appropriate. Middle consent implied that participants were told that their data might be shared with people working in specific research areas related to the study. Explicit consent implied that participants would be contacted for an opinion whenever there was a request for sharing.

Interviewees were generally in favor of broad or middle consent. Even when proposing broad consent, several suggested qualifying it by telling the participant about the sorts of recipients.

. . . Anticipate right at the beginning . . . who you are going to share it with . . . you actually say that I intend to share it with people working in similar institutes, working on infectious diseases . . . more or less identify my . . . dissemination breadth to the subject at the time of seeking consent . . . If . . . there is an unanticipated element of . . . data sharing . . . I would definitely go back to the patient . . . . (Senior Researcher, IN-SR-I-20, female)

One option was to include a list of organizations with whom data might be shared, possibly weighted toward organizations whose work could bring tangible benefits to participants. Broad consent would also reduce current concerns about overloading participant information sheets.

## Discussion

Both researchers and participants were generally in favor of data sharing as a means to increase evidence, transparency, value for money, and learning (Bersoff & Dawes, 1992; Bishop, 2009; Langat et al., 2011; Pisani & AbouZahr, 2010; Rani et al., 2011; Rani & Buckley, 2012; Tenopir et al., 2011). Their minimal experience of it, however, amplified a series of reservations (Pisani & AbouZahr, 2010). Underlying these was a narrative of powerlessness in the face of potential exploitation of participants by researchers (Mohammed, Walters, LaMarr, Evans-Campbell, & Fryberg, 2012), of data sharers by data accessors, and of researchers and institutions by more powerful researchers, institutions, and countries (Cooper, 2007; Tangcharoensathien, Boonperm, & Jongudomsuk, 2010). Our interviewees tended to adopt normative deontological rather than consequentialist ethical positions. Although their ideas about the potential benefits of data sharing tended to be utilitarian, concerns about moral duty and protection were more prominent. In this, they recapitulated the contemporary history of biomedical research ethics, with its emphasis on rectification of past harms to individuals. Protection of participants from (willful) harm was paramount for researchers closer to them, and protection of organizations and individual researchers was paramount for analysts and managers.

We saw some differences in emphasis between research participants, field-workers, and more senior researchers. Research participants emphasized the use of data to remedy problems their communities faced, but did not seem particularly concerned about consent in itself, provided that they felt that confidentiality would be maintained (Bersoff & Dawes, 1992). Field-workers tended to emphasize maintenance of trust and confidentiality (Bishop, 2009), the need for consent, and the imbalance between ease of access to “ready-made” data and the hard work they had put in to collect them. Recompense was expressed more in terms of benefits to communities than in the form of acknowledgment or authorship. Senior researchers were concerned about potential data massage, ownership, authorship, and managed access.

**Figure 1.**
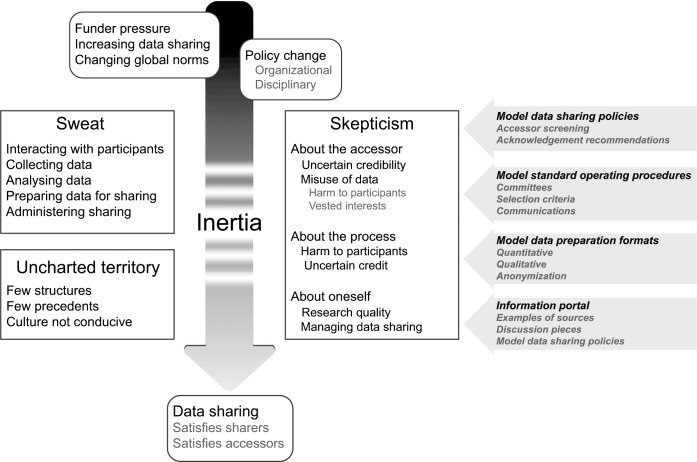
Themes emerging from analysis of opinions on data sharing among public health researchers and research participants.

Figure 1 illustrates the higher themes that emerged from our analysis. The international move toward data sharing for biomedical research needs to push through a syrup of inertia (Pisani & AbouZahr, 2010), to which we recognize three contributors: sweat, skepticism, and uncharted territory. We frame our discussion around them because we think that addressing them is central to any recommendations.

### Sweat

Researchers were often wary of external individuals using their data to write articles and reap benefits in terms of authorship and funding. One predictable reason for their wariness was the competitive nature of research, between both individuals and institutions. Perhaps as important, however, was a need for validation. Collecting data is hard. In public health research, it involves legwork, negotiation skills, an emotional commitment, and long hours, as well as the effort required to enter, clean, and prepare data for sharing (Chandramohan et al., 2008; Nelson, 2009; Pearce & Smith, 2011; Pisani & AbouZahr, 2010; Rani & Buckley, 2012; Savage & Vickers, 2009). The format and level of contextual and explanatory detail required to make qualitative data interpretable were undefined: “the inescapable problem of not being there” (Dicks, 2007). Vague ideas of what sharing would entail certainly contributed, as did doubt about the quality of one’s own datasets and position in a competitive hierarchy, but we think that uncertainty about personal and institutional validation was a central concern (Budin-Ljøsne et al., 2014).

### Skepticism

Skepticism about the process of data sharing was often expressed in terms of trust in the accessor, the process, or sharers themselves. There were substantial concerns that accessors might use data to harm participants or to meet vested interests (Cooper, 2007; Pearce & Smith, 2011). Uncertainty about their motives raised the possibility that use of secondary data might compromise participant anonymity, either individually or when collective data were used to represent an identifiable community and lead to stigma. This concern was marked when researchers were working on issues of social concern and when their institutions were involved in activism on behalf of vulnerable communities. For example, actors with a vested interest in clearing informal settlements might use information about their inhabitants to generate headcounts that could accelerate resettlement, or might use data on, for example, fertility, to criticize residents’ behaviors in the media. Conversely, the large numbers of people living in informal settlements are a potential target for products and services, and commercial interests might find the same sort of information useful financially (Anderson & Schonfeld, 2009; Budin-Ljøsne et al., 2014).

Misgivings about accessors’ motives and tangible benefits to both sharer and participants led to suggestions that potential accessors should be screened and established as credible. Credibility was determined by a researcher’s position in an institution and by institutional reputation. Reputability tended to assuage fears about harm to participants and misuse of data because it implied ethical research governance and established systems for data sharing that could be relied upon to protect participants. Interviewees showed both attraction to and misgivings about data sharing with northern organizations. On one hand, concerns about imbalance within the global order (and its familiar history) made them worry about exploitation (Harding et al., 2012; Tangcharoensathien et al., 2010). Researchers in lower-income settings “. . . want to move away from being primary producers of data for developed country scientists to analyze—they do not wish to remain hewers of data and drawers of protocols” (Chandramohan et al., 2008). On the other hand, they generally thought of multilateral organizations and northern universities as more credible than local ones, and their familiarity with India’s research culture made them perhaps more resistant to local data sharing. Collaboration with reputable international organizations might also add value by bringing data to a wider audience, generating international publications, boosting reputation, and opening up avenues for funding. It is possible, of course, that field-workers and community members who had limited familiarity with international research might have overrated the potential benefits of sharing data with institutions such as the WHO. For example, data collection for a multilateral agency might not lead directly to changes to health services for a community.

Skepticism about the process of data sharing often extended to researchers’ own capacity to do good research and collect data of good quality (Nelson, 2009; Savage & Vickers, 2009). Interviewees usually talked about data sharing from the point of view of the sharer, but when they did talk about data quality they would switch to the accessor’s point of view and express doubts about secondary uses of data that might be of inferior quality. Doubts were expressed about researchers’ ability to coordinate the process of sharing in an environment lacking established structures or about sharing with an accessor whose credibility had not been established to the sharer’s satisfaction.

### Uncharted Territory

Central to most interviews was confusion about what data sharing meant. It was confused with publication, distributing reports, sharing findings with the media, and dissemination among participants, and also with sample sharing for new laboratory analyses. Interviewees often struggled with this so much that we found time passing as we explained the idea and precedents. Some interviewees whom we had expected to be familiar with the issues surprised us with their confusion.

Our assessment is that much of the confusion arose because data sharing is more or less uncharted territory for most Indian researchers. There exist few precedents, few established structures, and little guidance (Tenopir et al., 2011), and the activities of institutions with experience are unknown to researchers outside them, all of this on a background of individual and organizational competition to conduct and publish high-value research within limited means and opportunities (Knoppers, Harris, Budin-Ljøsne, & Dove, 2014). The desire for validation fuels anxieties about a potential lack of it and an environment of territoriality and suspicion about accessors’ motives.

## Best Practices

Although our study focused on individuals associated with one organization, we think that the findings are generalizable, particularly in the sense that they represent an effort to engage with interviewees at each point along the chain from community participant to chief executive officer. Our suggestions for best practice are all about demystification and clarification. Research participants will be confident—and willing, we hope—to share data if the checks and balances in the process are understood and transmitted clearly to them by researchers they trust. To begin with, we need to chart the territory by providing clarity on how data sharing and consent should be explained to participants (Ioannidis, 2013; Nelson, 2009), how data should be prepared for sharing, how others should be alerted to their availability, how potential accessors should be screened and what their responsibilities are, and what individuals and organizations should expect in terms of validation, acknowledgment, and authorship (Rani & Buckley, 2012).

Included in Figure 1 are our suggestions for how to address the inertia between the desire to establish data sharing and its realization. We think that the inputs should be structural and include guidelines, protocols, and examples of good practice. For organizations, we need examples of data sharing policies that can be modified easily, and standard operating practices for the constitution of committees, selection criteria for accessors, and sequence of communications. We need examples of consent form text and of preparation formats for both quantitative and qualitative data (Dicks, 2007), including guidelines on anonymization. Finally, we need to make them available through a web-based resource that includes example sources and models for data sharing policies and data preparation.

## Research Agenda

Taking forward the data sharing agenda involves model behavior. We will make the qualitative data from our study available and attempt to disseminate guidelines and model policies and standard operating procedures. We can think of two ways in which research would be useful. First, a quantitative account of the ecological trend in data sharing in India, attempting to draw information from portals, institutions, and individuals on the rate of development of practice. Some of this could come from web metrics and some from proactive engagement with researchers. Second, a series of interviews with people who have not previously shared data, but go on to do so. It might be useful to generate a series of frequently asked questions about the processes, with answers based on the actual experiences of Indian researchers and research participants.

## Educational Implications

The educational imperative is to make resources available to researchers and managers across India and the world, with a sense of generalizability and credible imprimatur. To this end, we are developing model institutional data sharing policies, an open access online ethics toolkit that covers the concerns of potential sharers and accessors, and a training module that can be accessed online or nested within biomedical research ethics and governance courses.
